# Notes on the History of Anæsthesia

**Published:** 1893-12

**Authors:** James McManus

**Affiliations:** Hartford, Conn.


### NOTES ON THE HISTORY OF ANÆSTHESIA.[1]

BY DR. JAMES McMANUS, HARTFORD, CONN.[2]

At Greenfield last year a vote was passed requesting me to give an historical sketch of modern anæsthesia at this meeting, and in response to that invitation I will read to you a concise and condensed statement, relating incidents and experiments that led up to, and the facts regarding the discovery of, anæsthesia.

In looking up the records of the centuries we find mention made of various medicines that have been used for the purpose of

---

[1] Read at the Union Meeting of the Connecticut Valley Dental Society and the Connecticut State Dental Association at Hartford, Conn., May 16, 17, and 18, 1893.

[2] [While the principal facts connected with the discovery of anæsthesia have repeatedly been told and have become part of the history of the profession, the "Notes" by Dr. McManus seem to give to the story a new life, as they graphically depict the experiences of the time. The duty of keeping alive the memory of Dr. Wells remains with each generation, and it is therefore with unusual pleasure that we give space to this paper.—Ed.]

rendering patients insensible to pain while undergoing surgical operations.

Homer mentions the anæsthetic effects of nepenthe.

Dioscorides and Pliny allude to the use of mandragora.

Lucius Apuleius, who lived 125 A.D., and whose works were republished in the fourteenth century, says that if a man has to have a limb mutilated, sawn, or burnt, he may take half an ounce of mandragora wine, and whilst he sleeps the member may be cut off without pain or sense.

A Chinese physician who lived in the third century, named Hoa-tho, gave his patients a preparation of hemp, whereby they were rendered insensible during surgical operations.

The soporific effects of mandrake are mentioned by Shakespeare, as well as other draughts, the composition of which are not given.

After the lapse of centuries, Dr. B. W. Richardson, of London, procured specimens of mandragora root and had wine made from them, which he found by testing to have the properties ascribed to it by the ancient writers.

Theodoric, in a surgical treatise as early as the thirteenth century, gives directions how to prepare a "spongia somnifera" for inhalation before operations.

Dr. Simpson makes the statement that ether was known in the thirteenth century, that its formation was described in the sixteenth century by Valerius Cordus, and was first named "ether" by Frobenius in 1730.

A German work published in 1782 by Meissner mentions the case of Augustus, King of Poland, who underwent an amputation while insensible by the use of a narcotic.

Herodotus refers to the practice of the Scythians of inhaling the vapor of a certain kind of hemp to produce intoxication, and on September 3, 1828, M. Gerardin read to the Academy of Medicine of Paris a letter describing surgical anæsthesia by means of inhaling gases.

The ancients did know of and used the narcotics named, and possibly with all success that is claimed by these writers; but the compounds they made, and the manner of administering them, were not generally known outside their circle or territory, and after years their use was practically abandoned. Opium, seemingly, was the one drug that for many years prior to 1844 the surgeons mainly relied on to give relief from pain. It is on record that the ancient Greeks, Romans, and Arabians were familiar with and employed many volatile substances that they found acted more promptly when inhaled than when taken into the stomach, and after the discovery of oxygen by Priestley and Scheele in the middle of the last century, experiments were made with gases by physicians for the cure of diseases, especially of a consumptive type. A medical institute was established at Bristol, England, in 1798, and Sir Humphry Davy was appointed superintendent. While there he made a study of the effects of nitrous oxide, or laughing-gas, and after making many experiments with it, he made this remarkable statement early in 1800: "As nitrous oxide gas appears capable of destroying physical pain, it may probably be used to advantage during surgical operations in which no great effusion of blood takes place." He also stated that he had inhaled the gas and secured relief from pain while cutting a wisdom-tooth.

For more than forty years medical and scientific men were experimenting with the gas, the chemical and medical professors as well as popular lecturers gave entertainments with it, and always with watchfulness over their subjects for fear that in their excitement they might injure themselves, which they not infrequently did, and were not conscious of at the moment; and all these years the onlookers and lecturers could not see the great blessing that laughing-gas was offering to them, and tempting some one to lift to a nobler use than that of a mere means of amusement.

Professor G. Q. Colton gave a course of lectures on chemistry and natural philosophy in Hartford early in December, 1844; to popularize as well as amuse the audiences at these lectures the exhibition of the effects of laughing-gas on willing subjects was made a special feature of the entertainments. Dr. Horace Wells, well known in Hartford as a skilful dentist, attended with his wife the lecture given the evening of December 10, 1844. Dr. Wells inhaled the gas; the effect not being as pleasant as his wife wished for, she reproached him on the way home for taking it and making himself ridiculous before a public assembly. Dr. Wells went to that lecture to see, hear, and learn. He inhaled the gas, and subsequently watched its effects on others.

The exciting incident to him at the evening's entertainment was when Mr. Samuel A. Cooley, a well-known Hartford man, gave a lively exhibition of the effects of the gas by running and jumping about and falling, striking his legs against the wooden settees, and acting apparently perfectly unconscious of possible danger. After the effects of the gas had passed off, Dr. Wells asked him if he was not hurt, and he replied he did not know it at the time, but on looking at his legs found them bleeding from the injuries he had received. Dr. Wells, turning to Mr. David Clark, said, "I believe a man, by taking that gas, could have a tooth extracted or a limb amputated and not feel the pain."

Before leaving the lecture-hall Dr. Wells asked Mr. Colton whether one could not inhale the gas and have a tooth extracted without feeling any pain, and he replied that he had not given the subject any thought; that he had been giving the laughing-gas for over a year and such an idea had not occurred to him, and could not express an opinion. Dr. Wells then said that he was inclined to try the experiment on himself and have a troublesome tooth extracted if he would bring a bag of the gas to his office the next day. Late that evening Dr. Wells called on Dr. Riggs to tell him that he had attended the lecture of Professor Colton and with others had inhaled the gas, that Mr. Cooley had injured himself and was not conscious of it at the time, adding, "If he did not feel pain, why cannot the gas be used in extracting teeth?" A long discussion followed as to whether it would be right or safe for them to make such an experiment with possible danger staring them in the face, but Dr. Wells was so confident and fearless that he agreed to take the gas and have a tooth extracted the next day if Dr. Riggs would perform the operation. As requested, the next morning Professor Colton brought a bag of the gas to the office of Dr. Wells. There were present Professor Colton, Drs. Wells and Riggs, and as onlookers a Mr. Colton and Mr. Samuel A. Cooley, the star performer at the entertainment the night previous. Dr. Wells sat down in the operating-chair, took the bag into his hands and inhaled the gas until he was insensible, when Dr. Riggs extracted an upper wisdom-tooth. Dr. Wells remained unconscious a short time, and on recovering exclaimed, "I did not feel it so much as the prick of a pin. A new era in tooth-pulling. It is the greatest discovery ever made," and remarks of a similar nature, being, naturally, perfectly delighted with his successful experiment. The not improbable value of nitrous oxide gas, as suggested by Humphry Davy in 1800, proved a certainty December 11, 1844, when the first surgical operation was successfully performed on Dr. Horace Wells while under its influence. On that day modern anæsthesia was given to the world, and nitrous oxide gas proved to be a blessing to suffering humanity and the forerunner of all other anæsthetics.

I will read to you brief extracts from testimony given under oath before authorized officials, commencing with Professor Colton, who stated that Dr. Horace Wells came to him to learn how to prepare the gas, that he gave him full information, and advised him to go to Boston for necessary apparatus, as he could not furnish it. A few weeks after leaving Hartford he saw a paragraph in the papers announcing that Dr. Wells was extracting teeth without pain, and he stated on several occasions in connection with that paragraph how and when the discovery originated. Dr. J. M. Riggs testified that "We were so elated by the success of this experiment that we turned our attention to the extraction of teeth by means of this agent, and continued to devote ourselves to this for several weeks almost exclusively."

Dr. E. E. Marcy testified that while a student at Amherst College he had inhaled the gas, and also the vapor of sulphuric ether, and knew that the operation and effect of these substances were nearly similar, but he did not know that one or the other would produce insensibility to pain until Dr. Wells made the announcement. At the invitation of Dr. Wells he called at his office and witnessed the gas given and a tooth extracted, the patient showing neither excitement nor the slightest consciousness of pain. Dr. Marcy then suggested to Dr. Wells the use of sulphuric ether, his impression being that it possessed all the anæsthetic properties of the gas, was equally safe, could be prepared with less trouble, was less expensive, and could always be kept on hand. Dr. Marcy said he would prepare some ether and give him some of it, and also would make a trial of it himself in a surgical case that he expected to operate on in a few days. A few days later the ether was given to the patient alluded to, and an encysted tumor the size of an English walnut was cut from his head. Dr. Wells was present, the operation was successful, and conclusively proved the anæsthetic properties of ether vapor. Dr. Wells then told of a conversation held with Dr. Riggs regarding the effects of both ether and gas, and gave the opinion of Professor Rogers, of Washington (now Trinity) College, that the vapor of ether was much more dangerous than that of the gas.

"At the urgent request of Dr. Wells I read what I could easily procure in relation to both articles, and gave as my opinion that, as the gas was more agreeable and easy to inhale than the ether, it was, upon the whole, more safe, and equally efficacious as an anæsthetic." Dr. P. W. Ellsworth was also asked respecting the comparative safety of nitrous oxide gas and sulphuric ether, and he gave his opinion in favor of the gas, and advised Dr. Wells to confine himself to that agent. In the month of January, 1845, Dr. Wells went to Boston for the purpose of making known his discovery. He obtained permission of the elder Dr. Warren to address his class in the medical college, and at the close of his talk he gave the gas to a boy and extracted a tooth. The boy screamed out, but on recovery said he did not know when the tooth was drawn. The students hissed and cried humbug. They were not willing to treat seriously any attempt to investigate the anæsthetic properties of the gas.

Dr. William T. G. Morton had been a student of dentistry with Dr. Wells in 1841 and 1842, but was living in Boston at this time, and renting an office of Dr. C. T. Jackson.

In conversation with these gentlemen, they both tried to discourage him, having no faith in his statements, and advised him to give up the use of the gas. Dr. Jackson, noted then as a chemist, treated the subject as lightly as did the medical students, calling it a humbug. That a dentist from a country town could appear in Boston and announce to the world that he had made such a grand discovery was not to be credited, and Dr. Wells soon learned that not one of the influential medical or scientific men in that learned city could be induced to interest themselves in investigating the properties of the gas or lend him any assistance whatever while he remained in that city. He returned to Hartford greatly depressed and in poor health, but in a short time was able to resume his practice. During that and the following year he continued to give the gas freely, and when not able from any cause to attend the patients, he would bring or send them to the office of Dr. Riggs to have him give the gas.

In the *Boston Medical and Surgical Journal* of June 18, 1845, there was an article written by Dr. P. W. Ellsworth, of Hartford, Connecticut, on the "Modus Operandi of Medicine," in which he mentions that the nitrous oxide gas has been used in a number of cases by our dentists, and has been found to perfectly destroy pain, and no unpleasant effects follow its use. Dr. Morton visited in Hartford during the summer of 1845, and called with Dr. Wells on Dr. Riggs to talk about the gas, and wanted them to give him some, and tell him how it was prepared. Dr. Wells referred him to Dr. Jackson, of Boston, who he said could prepare it for him, or tell him how it should be done, as he knew all about it.

In the summer of 1846, Miss Elizabeth Williams, of Hartford, met Dr. Morton in Stafford Springs, Connecticut; learning that he was a dentist, she told him her experience with the laughing-gas, and that Dr. Wells had extracted a tooth for her on the 6th of March, 1845. He asked her about the effect and operation of the gas, and gave no intimation to her that he had any knowledge of the gas or of any other anæsthetic. Drs. Wells, Riggs, and Terry, of Hartford, continued to give the gas in their practice with success, and great was their surprise when they learned that Drs. Jackson and Morton were heralded in the Boston papers in the fall of 1846 as the discoverers and the inventors of a compound which they stated by breathing into the lungs induced so deep a slumber as to enable them to perform the most painful surgical operation with entire unconsciousness on the part of the patient.

Dr. Morton made his so-called discovery September 30, 1846, when he extracted a tooth for Mr. Eben Frost under the influence of his pretended compound.

He made known the result of his experiments to Dr. Jackson, and they found, as Drs. Marcy and Wells, of Hartford, had demonstrated nearly two years earlier, that by inhaling the vapor of sulphuric ether it would produce unconsciousness, and surgical operations could be performed without pain while under its influence.

Soon after, he called on Dr. Warren, who promised him an early chance to test his compound, and on the 16th of October, 1846, he made his first experiment at the hospital, and on the 17th another, both surgical cases.

Boston surgeons were at last convinced that anæsthesia had been discovered, and Boston men were the discoverers. The managers of the Massachusetts General Hospital were now ready to claim for their institution the honor and credit of first demonstrating this great fact to the world, and Boston surgeons, Boston newspapers, and the public were now very much interested, and only too ready and anxious to assist the assumed discoverers in introducing their pretended discovery and advising its use in general surgery. Dr. Morton wrote to Dr. Wells, October 19, telling him of his discovery, stating that he had patented it, and wishing to know if he would not like to visit New York and sell rights to use it. Dr. Wells replied to that letter, October 20, that he would be in Boston soon, and he and his wife took an early train the Saturday after, arriving in Boston about mid-day. After dinner he called on Dr. Morton, remaining with him about two hours. On his return Mrs. Wells asked him if Dr. Morton had discovered anything new, and he replied, "No; it is my old discovery, and he does not know how to use it." He said he perceived what it was on entering his room; he knew it was nothing but ether. On being asked if he would assist in selling his patent rights, he replied, "No, he would have nothing to do with him."

Dr. Wells and wife returned home on the following Monday.

The statement made in the letter of October 19 to Dr. Wells, that he had patented his compound, was not true, and at the interview a few days later, in Boston, it did not occur to him that Dr. Morton intended to deprive him of the credit of the original discovery, but that he did claim the discovery and application of a new and more convenient agent. The possible money value that might accrue to them from a vigorous pushing of the discovery set the doctor and dentist to figuring out futures. They decided to apply for a patent, which the Patent Office records say was done in the names of Drs. C. T. Jackson and W. T. G. Morton, October 27, 1846; but before the patent was granted, Dr. Jackson, fearing he might be censured or even expelled from the Massachusetts Medical Society if he took out a patent, made an assignment, which apparently gave to Dr. Morton all his right, title, and interest in the then assumed invention, but for which act he obligated Dr. Morton to pay him ten per cent. of all he made out of it, and later on, through his counsel, he demanded twenty-five per cent. of all the profits both at home and abroad, which Dr. Morton refused to give.

The patent was granted November 12, 1846. Circulars were printed with the names of Drs. Jackson and Morton as the discoverers and inventors of a compound that later proved to be the well-known fluid sulphuric ether, and they were distributed broadcast. Agents were sent out to sell rights. The doctor, dentist, or anybody, qualified or not, who would pay the price, could buy the right to use this wonderful and powerful agent.

The scale of prices being, for cities of over one hundred and fifty thousand inhabitants, two hundred dollars; fifty thousand and under, one hundred and fifty dollars; cities under five thousand, thirty-seven dollars, for a term of seven years.

The following advertisement was published in the *Boston Evening Traveller* of November 29, 1846, signed by Drs. N. C. Keep and Wm. T. G. Morton:

"The subscribers, having associated themselves in the business of dentistry, would respectfully invite their friends to call on them at their rooms, No. 19 Tremont Row. They confidently believe that the increased facilities which their united experience will afford them of performing operations with elegance and despatch, and the additional advantage of having them performed without pain, by the use of the fluid recently invented by Drs. Jackson and Morton, will not only meet the wishes of their former patients, but secure to them additional patronage."

This was a unique appeal to the Boston citizens for patronage, equalling, if it does not far surpass, many of the advertisements that are to be seen in the newspapers of our day.

The physician or dentist that indulges in a much less flagrant style of advertising to-day is barred out of both medical and dental societies.

Soon after the extraction of the tooth for Mr. Frost by Dr. Morton, Dr. Jackson sent a letter to a friend in Paris, France, giving the particulars of his pretended discovery, stating that he had persuaded a dentist in Boston to administer the vapor of sulphuric ether to his patients when they wished to have teeth extracted, and they suffered no pain during the operation; and later a second letter, stating that it had been used in the Massachusetts General Hospital with great success. These facts he wished his friend to communicate to the Paris Academy of Sciences. Soon after the letters were sent there was a falling out between the Boston discoverers. The public then learned from their controversy of the bitter feeling existing, and found, also, that each one denied that the other had any just claim for the credit of the discovery.

The Paris Medical Institute, in response to the letters sent by Dr. Jackson, and with the knowledge only of his claim and that of Dr. Morton, awarded to each one the sum of two thousand five hundred francs; to Dr. Jackson for the discovery of the principle, and to Dr. Morton for the application of it. The Institute at the time knew nothing of the claims of Dr. Wells. While the controversy was going on so bitterly in Boston, Dr. Wells decided, partly on account of his health, to take a trip to Europe, and while there to interest, if possible, and to present his claim as the discoverer of anæsthesia to the English and continental surgeons. While in Paris he made the acquaintance of the American dentist Dr. Brewster, through whose good influence the subject was again and properly brought before the French Academy of Medicine. On Dr. Wells's return to this country he found the influence of medical and scientific men, the professional journals, and newspapers were all in favor of sulphuric ether, and the tide running in favor of the claims of Drs. Jackson and Morton.

Late in the year 1847 a new agent, chloroform, was introduced by Professor James Y. Simpson, M.D., of Edinburgh, Scotland, and that for a time seemed likely to supplant sulphuric ether. Dr. Wells gave the nitrous oxide gas on January 1, 1848, to Henry A. Goodale, and Dr. P. W. Ellsworth amputated his leg. Also, January 4, gave the gas to Mrs. Gabriel, and Dr. S. B. Berresford removed a fatty tumor from her right shoulder. Dr. Wells later in the month went to New York to visit the hospitals and to urge his claims with the surgeons in that city. The worry, annoyance, and injustice done him by the rival claimants, increased by the experiments he was making with different anæsthetic agents, brought on serious mental disturbance, and under these influences, disheartened and despondent, he put an end to his sufferings, January 24, 1848.

The following letter arrived soon after his death from his friend Dr. Brewster, dated

"PARIS, January 12, 1848.

"MY DEAR WELLS,—I have just returned from a meeting of the Paris Medical Society, where they have voted that to Horace Wells, of Hartford, Connecticut, United States of America, is due all the honor of having first discovered and successfully applied the use of vapors or gases whereby surgical operations could be performed without pain. They have done even more, for they have elected you an honorary member of their Society. This was the third meeting that the Society had deliberated upon the subject. On the two previous occasions Mr. Warren, the agent of Dr. Morton, was present and endeavored to show that to his client was due the honor, but he, having completely failed, did not attend the last meeting. The use of ether took the place of nitrous oxide gas, but chloroform has supplanted both, yet the first person, who first discovered and performed surgical operations without pain, was Horace Wells, and to the last day of time must suffering humanity bless his name.

"Your diploma and the vote of the Paris Medical Society shall be forwarded to you. In the interim you may use this letter as you please.

"Believe me ever truly yours,

"BREWSTER."

Drs. Jackson and Morton from the start had persistently stated that the nitrous oxide gas was a failure; that it was not an anæsthetic; and they also as persistently ignored the fact that Drs. Wells and Marcy had used sulphuric ether with success, but had decided, in consultation with Dr. Ellsworth, that as the gas was more pleasant and agreeable to take, as well as less dangerous, it would be better to continue its use in dental operations. The death of Dr. Wells left the field open for them, and as the new agent, chloroform, was making a very successful record, it soon became so popular that the use of gas was given up and by many forgotten.

Hartford had no medical school, hospital surgeons of national reputation, or professional journals to compete with Boston, that had all these advantages, while the great influence of Boston surgeons, Boston journals, and Boston wealth were freely given to aid the Boston claimants in their attempt to rob Dr. Wells of the honor and credit of his discovery. Boston influence aided them in their successful appeals to the rich and the profession for remuneration, and Boston money helped them in wining and dining a memorable lobby influence in its attempts to get through Congress a bill granting them one hundred thousand dollars for the use of their pretended discovery by the army and navy. Through the efforts of the Hon. Truman Smith, United States senator, and the members of Congress from Connecticut the passage of this bill was defeated.

Soon after the introduction of chloroform, and the death of Dr. Wells, the use of the gas was abandoned, but the surgeons and the public were soon taught that with chloroform and ether they were dealing with uncertain and dangerous agents. The frequent deaths reported, and the ill effects that often followed their use, caused a feeling of dread on the part of both patient and operator, so that comparatively few cared or dared to risk taking or giving either of them.

From 1848 until 1863 the longing for a safe anæsthetic was universal; again, fortunately for suffering humanity, Professor Colton appears before the public as a lecturer and exhibitor of laughing-gas, and at a private entertainment to a specially invited party in New Haven, June, 1863, he related the history of the discovery of anæsthesia by Dr. Wells, stating that since that time he never had been able to induce a dentist to try the gas. Our old friend, and for many years treasurer of this Society, Dr. J. H. Smith was present, and then offered to try the gas again if Professor Colton would administer it. The professor said he would be very glad to do it, as he wished to again demonstrate what could be done with the gas. Their first patient was an old lady, for whom they extracted seven teeth; after recovering from the effects of the gas, she was so pleased with the result that she allowed Professor Colton to announce to his next audience her name and that she had had seven teeth extracted without pain, and without any ill or unpleasant effects from the gas. In three weeks and two days from that time Drs. Smith and Colton extracted over three thousand teeth.

Their success induced Professor Colton to abandon the exhibition business and to establish the Colton Dental Association in Cooper Institute, New York, devoted exclusively to the extraction of teeth with the gas.

In a pamphlet published by Dr. Colton in 1866 he says, "Whatever credit I deserve in connection with this matter is derived from the fact that I revived the use of gas, after it had been condemned, dead and forgotten as an anæsthetic from 1848 to 1863. In this revival and demonstration of the value of the gas as an anæsthetic is not the world practically indebted to me for its use? If I had not revived it, by whom would it have been done? That poor Wells failed to convince the world of its value does not militate in the slightest degree against the honor he deserves as the discoverer of anæsthesia. He did all that a man could do under the circumstances."

Dr. Colton's great faith, and the co-operation and good work done by Dr. Smith, encouraged the dental profession to again take up the use of the gas, and the uniform success with it proved again to the public the true value of the gas as the original, safe, and reliable anæsthetic. Eighteen years after the death of Dr. Wells, there appeared in the *New York Medical and Surgical Reporter* of January 6, 1886, a report made by Dr. J. M. Carnochan, chief of staff in the New York State Emigrant Hospital, of three surgical cases that he performed, the patients being put under the influence of nitrous oxide gas by Dr. Colton, and February 10 of the same year he reported four more operations upon adults, making in all seven successful capital operations under the influence of the gas. After the first operations he said, "I have no hesitation in stating that the nitrous oxide gas as an anæsthetic is far superior to either chloroform or ether; the operation being attended by no nausea or sickness, and without the dangerous effects often incident to chloroform and ether. It is not improbable that had Wells lived and had the boldness to follow up his early successful experiments, chloroform and ether would never have been thought of as anæsthetics." In Dr. Carnochan's second report, giving a *résumé* of seven capital operations under the influence of nitrous oxide gas, he says, "I have also during this time used chloroform and ether in many operations, and my opinion in regard to the superiority of the nitrous oxide gas as an anæsthetic is still unchanged. I believe, however, that there is great room for improvement in the mode of administration of the gas."

The success attending the revival of the use of the gas, and the testimony given by the surgeons in New York and elsewhere, was simply a repetition of the success attained by Dr. Wells while he was alive and able to attend to his practice in Hartford.

The General Assembly of Connecticut in 1847 passed resolutions in favor of Dr. Wells as the discoverer of anæsthesia, and declared that he was entitled to the favorable consideration of his fellow-citizens, and to the high station of a public benefactor. The Court of Common Council of the city of Hartford passed resolutions to the same effect. The physicians and surgeons of the city united in a testimonial declaring their belief in the justice of the claims of Dr. Wells. The Paris Medical Society, January, 1848, voted that to Dr. Horace Wells, of Hartford, Connecticut, is due all the honor of having first discovered anæsthesia.

The testimony of Professor Valentine Mott, M.D., of New York, December 20, 1852, is that Dr. Wells is entitled to the credit and honor of the discovery. Professor R. D. Mussey, of Cincinnati, Ohio, in a letter to the Hon. Truman Smith, United States senator from Connecticut, December 24, 1852, says, "I have long regarded Dr. Wells as entitled to the credit, and to the pecuniary award if any such consideration is to be made, for the invaluable discovery of anæsthesia."

Dr. C. H. Haywood, who was house surgeon in the Massachusetts General Hospital at the time Dr. Morton administered his pretended compound there, in a letter to United States senator Truman Smith, concludes with these words: "But before all let full and ample justice be done to that noble genius which first conceived the grand idea which has been the basis of all the experiments and the father of all the discoveries. To the spirit of Dr. Horace Wells belongs the honor of having given to suffering humanity the greatest boon it ever received from science."

In the early days it was difficult to prepare the gas in large quantities or to keep it on hand any length of time. Soon after the revival of its use in 1863 many improvements were made in apparatus for making gas, and, later, when the process was so perfected that dealers could furnish the gas to the profession in liquid form, in iron cylinders holding from one hundred to fourteen hundred gallons to be used from as desired, without danger of waste, loss of power or purity, all the former objections to its use were removed.

If Dr. Wells had been a resident of Boston, an M.D., and a member of the staff of the Massachusetts General Hospital, his discovery in 1844 would have been quickly accepted. As a stranger and a dentist, his claim as a discoverer and the evidence he had to sustain it, as well as the prediction made by Sir Humphry Davy many years before as to the probable properties of the gas, could not awaken enough interest in the minds of the stupid, stubborn, and jealous men that he appeared before to induce them to make another trial of the gas. They condemned it as a humbug, and suffering humanity was deprived of the blessing of an agreeable and safe anæsthetic for over twenty years.

Professor S. D. Gross, of Philadelphia, some years ago, when speaking before the American Medical Association, said that "Dentistry is the most important specialty in medicine: many people come into the world, and go out of it, who never require the services of other specialists; but no child is born who does not sooner or later require the services of a dentist." Terse and true as this statement is, equally true is the statement that modern anæsthesia, in all the varied modes of its administration, is undeniably the result of a dentist's heroic experiment and discovery. It is also sadly true that it was two years after the discovery, and after repeated successful operations in the hands of Hartford dentists, before Boston surgeons could be induced to accept the fact that an anæsthetic had been discovered. The record is now well up in the millions of successful operations made while under the influence of the gas, with evidence accumulating daily, all over the world, that the gas is a safe and reliable anæsthetic, and with abundant testimony to prove that Dr. Wells was the first to submit to a surgical operation while under its influence. These facts cannot be blotted out by the efforts of magazine writers that either ignorantly or wilfully ignore, as does the inscription on the ether monument that stands in the public garden in the city of Boston, the claims of Dr. Horace Wells.

The monument in Boston commemorates the discovery of anæsthesia by inhalation of ether as first proved to the world at the Massachusetts General Hospital, October, 1846. It is a beautiful work of art, with bas-relief pictures that tell to the onlooker the great blessing that some one had given to suffering humanity. The inscription tells an untrue story, and the stranger seeks in vain for the name of the world's great benefactor.

On Bushnell Park, in Hartford, there stands a monument, erected by the State of Connecticut and the city and citizens of Hartford, commemorating this great discovery of anæsthesia first given to the world in Hartford, in 1844, with the name inscribed, and a portrait statue of Dr. Horace Wells, to whom alone belongs the honor of its discovery, and who gave it to the world, to be as free as the air we breathe.

It will be remembered that Dr. Carnochan was decided in his opinion as to the value of the gas in surgical operations. He reported in January and February, 1866, seven successful operations, one case being the removal of an entire breast and the glands of the axilla, for cancer. The patient was a lady in feeble health, suffering from disease of the throat and lungs, and general debility. In thirty-five seconds from the time she began inhaling the gas she was in a profound anæsthetic sleep. She remained insensible for sixteen consecutive minutes, until the operation was completed, and, in forty seconds from the time the bag was removed, awoke to consciousness without nausea, sickness, or vomiting. With the added testimony of leading surgeons in New York, that the gas was the safest of all the anæsthetics, and with all the improvements for administering it, as also the certainty of being able to get and keep it on hand pure, is it not strange, to state it mildly, that surgeons should so persistently ignore the gas, and continue to give chloroform, ether, or both in combination, when about to perform simple operations, despite the fatal record that has so often given publicity to that class of cases? Dentists have proved the value of nitrous oxide. In years to come, it is barely possible, the surgeons may again wake up to its value and safety.

